# Causal effects of gut microbiota on appendicitis: a two-sample Mendelian randomization study

**DOI:** 10.3389/fcimb.2023.1320992

**Published:** 2023-12-15

**Authors:** Zehui Wang, Lijie Bao, Lidong Wu, Qi Zeng, Qian Feng, Jinchuan Zhou, Zhiqiang Luo, Yibing Wang

**Affiliations:** ^1^ Department of Emergency, The Second Affiliated Hospital, Jiangxi Medical College, Nanchang University, Nanchang, Jiangxi, China; ^2^ Queen Mary University of London, Nanchang University, Nanchang, Jiangxi, China

**Keywords:** Mendelian randomization, appendicitis, gut microbiota, causal inference, genetics

## Abstract

**Background:**

Previous research has posited a potential correlation between the gut microbiota and the onset of appendicitis; however, the precise causal connection between appendicitis and the gut microbiota remains an unresolved and contentious issue.

**Methods:**

In this investigation, we performed a Mendelian randomization (MR) analysis employing publicly accessible summary data extracted from genome-wide association studies (GWAS) to elucidate the potential causal nexus between the gut microbiota and the development of appendicitis. We initially identified instrumental variables (IVs) through a comprehensive array of screening methodologies, subsequently executing MR analyses using the Inverse Variance Weighted (IVW) technique as our primary approach, supplemented by several alternative methods such as MR Egger, weighted median, simple mode, and weighted mode. Additionally, we implemented a series of sensitivity analysis procedures, encompassing Cochran’s Q test, MR-Egger intercept test, Mendelian Randomized Polymorphism Residual and Outlier (MR-PRESSO) test, and a leave-one-out test, to affirm the robustness and validity of our findings.

**Results:**

Our investigation indicates that an elevated prevalence of Deltaproteobacteria, Christensenellaceae, Desulfovibrionaceae, Eubacterium ruminantium group, Lachnospiraceae NK4A136 group, Methanobrevibacter, Desulfovibrionales, and Euryarchaeota is inversely associated with the risk of appendicitis. Conversely, we observed a positive correlation between an increased abundance of Family XIII, Howardella, and Veillonella and the susceptibility to appendicitis. Sensitivity analyses have corroborated the robustness of these findings, and Mendelian randomization analyses provided no indications of reverse causality.

**Conclusion:**

Our Mendelian randomization (MR) analysis has unveiled potential advantageous or detrimental causal associations between the gut microbiota and the occurrence of appendicitis. This study offers novel theoretical and empirical insights into the understanding of appendicitis pathogenesis, along with its implications for preventive and therapeutic strategies.

## 1 Background

Acute abdominal pain constitutes 7%-10% of all emergency department visits (Cervellin et al., 2016), with appendicitis emerging as the leading cause for individuals seeking emergency medical care due to abdominal distress (Di Saverio et al., 2020). Moreover, appendicitis stands as a prevalent cause of acute abdominal pain necessitating surgical intervention (Munakata et al., 2021). The lifetime risk of acute appendicitis encompasses 8.6% for males and 6.7% for females (Addiss et al., 1990), with indications suggesting a rising incidence of appendicitis in industrialized nations (Ferris et al., 2017). Appendicitis can be categorized into two forms: complicated and uncomplicated appendicitis (Perez and Allen, 2018). Primary etiological factors underlying appendicitis encompass appendiceal lumen obstruction, lymphoid hyperplasia, and infections (Vanhatalo et al., 2019). The etiology of appendicitis includes bacteria, fungi, viruses, parasites, etc. (Yigiter et al., 2007; Katzoli et al., 2009; Larbcharoensub et al., 2013; Altun et al., 2017; Habashi and Lisi, 2019; Jones et al., 2023). Presently, surgical intervention remains the primary treatment modality for appendicitis(Di Saverio et al., 2020), albeit mounting evidence supporting the use of antibiotics as the first-line treatment for most cases of uncomplicated acute appendicitis (Salminen et al., 2015; Sallinen et al., 2016; Podda et al., 2019). This underscores the potential feasibility of non-surgical approaches in the management of appendicitis. Urgent endeavors are warranted to delve deeper into the etiological aspects of appendicitis in order to explore novel avenues for its prevention and therapeutic intervention.

The term “gut microbiota” encompasses the complex microbial community residing within the human intestinal tract, encompassing bacteria, fungi, viruses, and more. It has been established that the gut houses a staggering count of up to 100 trillion symbiotic microorganisms, with a cellular abundance tenfold greater than that of the human body itself (Backhed et al., 2005). The gut microbiota assumes pivotal roles within the human system, encompassing the enhancement of immune system functionality, integral contributions to digestion and metabolic processes, modulation of epithelial cell proliferation and differentiation, mitigation of insulin resistance, and influence on insulin secretion, among other functions (Gomaa, 2020).

Research has unveiled distinctions in the gut microbiota profiles between appendicitis-afflicted individuals and their healthy counterparts. Specifically, appendicitis patients have exhibited diminished richness and diversity in Firmicutes, Actinobacteria, Fusobacteria, and Verrucomicrobia (Peeters et al., 2019). However, conflicting findings have emerged, with certain studies reporting an elevated abundance of Fusobacterium and a decreased presence of Bacteroides in samples from individuals with appendicitis (Zhong et al., 2014). Likewise, discernable differences have been noted in the diversity and composition of the gut microbiota between cases of uncomplicated and complicated appendicitis (The et al., 2019). These investigations collectively suggest a plausible connection between the gut microbiota and the development of appendicitis, yet the precise causal relationship remains a subject of ongoing debate and uncertainty.

Mendelian randomization (MR) stands as an epidemiological approach that leverages genetic variations as instrumental variables (IVs) to infer causal links between exposures and outcomes. In contrast to conventional observational studies, MR has the capacity to mitigate confounding factors and reverse causation, thus establishing robust causal connections. Single nucleotide polymorphisms (SNPs), distributed randomly at conception and independent of confounding influences, render Mendelian randomization (MR) akin to randomized controlled trials, thereby circumventing the biases inherent in observational studies. Notably, no prior investigations have undertaken the assessment of the causal implication of the gut microbiota in appendicitis risk through MR analysis. In this research endeavor, we have undertaken a two-sample MR analysis utilizing summary statistics data derived from genome-wide association studies (GWAS) to scrutinize the interplay between the gut microbiota and appendicitis, thereby contributing to an enhanced understanding of its pathogenesis. This study offers fresh theoretical and empirical evidence pertinent to appendicitis prevention and treatment.

## 2 Methods

### 2.1 Study design and data sources

**Figure 1:**
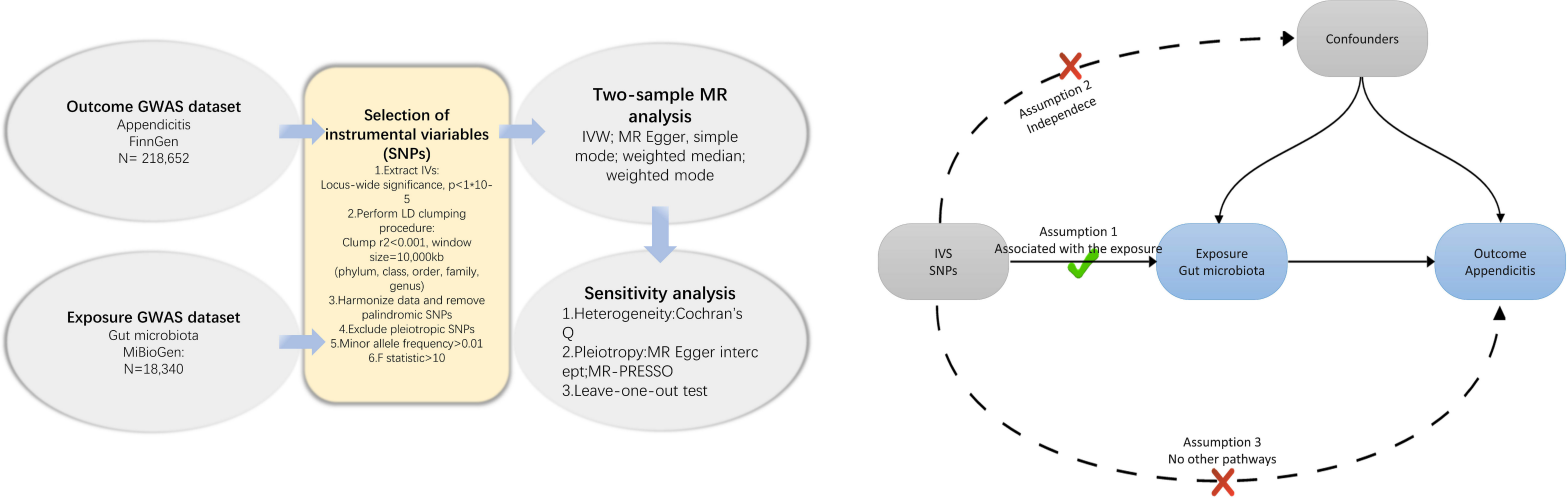
Flowchart of the present MR study and major assumptions. MR, Mendelian randomization; GWAS, genome-wide association study; SNPs, single nucleotide polymorphisms; IVW, inverse-variance weighted; LD, linkage disequilibrium; MR-PRESSO, MR pleiotropy residual sum and outlier.

We conducted a Mendelian Randomization (MR) study to explore the causal relationships between the gut microbiota and appendicitis The schematic representation of our research process is depicted in **Figure 1**. In summary, we identified genetic variants associated with the exposure by extracting data from Genome-Wide Association Study (GWAS) summary statistics, which were subsequently utilized as instrumental variables (IVs). We performed a sequential two-sample MR analysis employing five distinct MR methodologies. Finally, a comprehensive set of sensitivity analysis metrics, including tests for heterogeneity, pleiotropy, and leave-one-out analysis, were applied to assess significant associations.

Summary-level genomic data of the gut microbiota were acquired from the MiBioGen study (Kurilshikov et al., 2021; Consortium, 2023). This study represented the largest and most diverse genome-wide meta-analysis of the gut microbiota to date, encompassing genome-wide genotyping data and 16S fecal microbiota profiles from 24 cohorts, comprising a total of 18,340 individuals. The majority of participants in the study were of European descent (N=13,266). Profiling of microbial composition was achieved through targeted sequencing of the V4, V3-V4, and V1-V2 regions of the 16S rRNA gene. Subsequently, taxonomic classification was performed utilizing direct taxonomic binning. Following the processing of 16S microbiome data, a total of 211 taxa were identified, encompassing 131 genera, 35 families, 20 orders, 16 classes, and 9 phyla. Comprehensive information regarding the microbiota dataset can be found in the original investigation (Kurilshikov et al., 2021).

The summary GWAS data for appendicitis comes from FinnGen,which includes 16766 appendicitis patients and 201886 controls, with a total of 16380466 SNPs, all of whom are of European ancestry (Kurki et al., 2023). We conducted a search on the “ieu open gwas project” website using the keyword “appendicitis”. After considering our research needs, we decided to select the most recent and largest sample size dataset, called “Appendicitis, broad definition (Dataset: finn-b-APPENDICITIS BROAD)”. This dataset contains a comprehensive range of data related to appendicitis, which will provide a more comprehensive basis for our analysis in our research (Ben et al., 2020; Bristol, 2023).

### 2.2 Instrumental variables selection

To ensure the accuracy and validity of our conclusions regarding the causal relationship between gut microbiota and appendicitis risk, we implemented a series of quality control procedures to filter instrumental variables (IVs). Firstly, we selected single-nucleotide polymorphisms (SNPs) with significant associations to the gut microbiome as IVs. SNPs were chosen based on two distinct thresholds. In order to obtain a comprehensive overview and enhance the explained phenotypic variability, we included a set of SNPs with locus-wide significance levels below 1×10^-5^ as IVs. Additionally, No SNPs with genome-wide significance (*p*<5×10^-8^) was found in our study, therefore no secondary analysis was conducted using SNPs with genome wide significance. Secondly, to ensure the independence of the selected IVs and minimize the impact of linkage disequilibrium that violates the random allele assignment, we configured the clumping procedure with parameters set to r^2^<0.001 and kb=10,000kb. Thirdly, If exposure-related SNPs were not identified in the outcome genome-wide association study (GWAS) results, proxy SNPs highly correlated with the target variant (r^2^>0.8) were identified through the SNiPA website (Arnold et al., 2015). However, it’s important to note that such a scenario did not occur in our analysis. Fourthly, SNPs with palindromic properties and incompatible alleles were disqualified from the Mendelian Randomization (MR) analysis. Fifthly, in order to satisfy the second key assumption of MR (independence from confounders), we conducted a manual inspection and exclusion of SNPs significantly associated (p<5×10^-5^) with potential confounding factors using the PhenoScanner GWAS database (Kamat et al., 2019). No SNPs that may be significantly associated with potential confounding factors were found. Sixthly, a minimum minor allele frequency threshold of 0.01 was enforced. Lastly, to mitigate weak instrumental bias, the F-statistic was computed for each SNP (Burgess and Thompson, 2011), and any SNPs with F-statistics below 10 were discarded. The F-statistic is expressed as R^2^(n-k-1)/k(1-R^2^), with n representing the sample size, k denoting the number of IVs, and R^2^ signifying the variance explained by the IVs.

### 2.3 Effect size estimate

We conducted a two-sample Mendelian randomization (MR) analysis to explore the causal relationship between gut microbiome features and the risk of appendicitis When multiple IVs were involved in a gut microbiota feature, we adopted the inverse-variance weighted (IVW) test as the primary analytical approach, complemented by other methodologies, including MR-Egger, simple mode, weighted median, and weighted mode (Burgess et al., 2013). To comprehensively assess the influence of the gut microbiome on appendicitis risk, the meta-analysis technique known as IVW converted the outcome effects of IVs on exposure effects into a weighted regression model with an intercept constrained to zero. In the absence of horizontal pleiotropy, IVW yielded unbiased estimates by mitigating the influence of confounding variables (Holmes et al., 2017). It is noteworthy that the MR-Egger method may be susceptible to the influence of outlier genetic variables, potentially leading to incorrect estimations. However, even when all selected IVs are invalid, the MR-Egger approach can still produce unbiased estimates (Bowden et al., 2016b). The simple mode offers robustness against pleiotropy effects, although it may be less statistically powerful than IVW (Milne et al., 2017). The weighted median method, when at least 50% of data from valid instruments are available, is capable of providing precise and reliable effect estimates (Bowden et al., 2016a). In situations involving genetic variables that violate the pleiotropy assumption, the weighted mode method can be adapted (Hartwig et al., 2017).

### 2.4 Sensitivity analysis

To assess the potential impact of heterogeneity and pleiotropy among instrumental variables (IVs) on MR results, a comprehensive set of sensitivity analyses was undertaken to ascertain the robustness of our significant findings. Heterogeneity among the selected genetic instruments was quantified using Cochran’s Q test and visualized through funnel plots. Furthermore, we probed for potential horizontal pleiotropic effects of the included IVs, employing both the MR Egger intercept and the Mendelian randomization pleiotropy residual sum and outlier (MR-PRESSO) global test. Concurrently, we performed a leave-one-out sensitivity analysis to validate the precision and robustness of causal effect estimates, ensuring that our MR estimates were not unduly influenced by highly influential SNPs. In addition, the MR Steiger directionality test was employed to infer the causal direction (Hemani et al., 2017). Credible causal links were identified when the variance explained by the IVs on the exposure exceeded that on the outcome. All statistical analyses in our investigation, encompassing both MR and sensitivity analyses, were executed using the R packages “TwoSampleMR” and “MRPRESSO” within the publicly available R software (version 4.3.1).

## 3 Results

Utilizing the aforementioned criteria for instrumental variable (IV) selection, we identified 113 single nucleotide polymorphisms (SNPs) with a significance level of *p*<1×10^-5^ that exhibited substantial associations with gut microbiota at various taxonomic levels, encompassing class, family, genus, order, and phylum, which we subsequently employed as IVs. Detailed information regarding these selected SNPs, including effective alleles, alternative alleles, β values, standard error (SE) values, and p-values, is available in the **Supplementary Material** (see **Supplementary Table 1**).

To satisfy the second pivotal assumption of Mendelian randomization (MR), which necessitates independence of the instrumental variables (IVs) from confounding variables, we meticulously examined these SNPs using the PhenoScanner GWAS database (Staley et al., 2016; Kamat et al., 2019). We systematically excluded SNPs that displayed significant associations (*p*<5×10^-5^) with potential confounding factors. It is noteworthy that we did not detect any SNPs exhibiting links to confounding variables. Furthermore, all the chosen instrumental variables (IVs) boasted F-statistics exceeding 10, signifying the absence of evidence for weak instrument bias.

**Figure 2:**
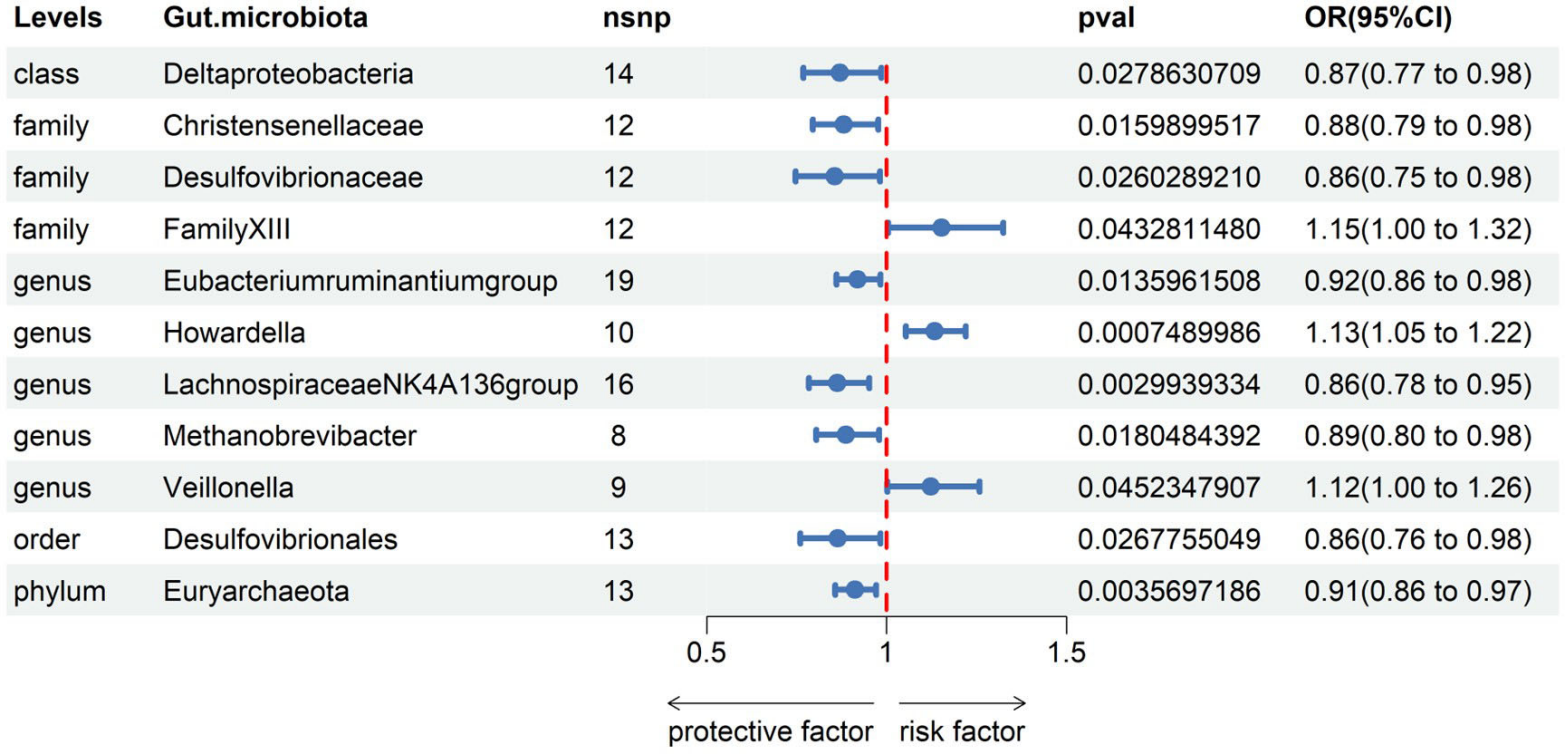
Associations of genetically gut microbiota with appendicitis risk using IVW methods SNPs, single nucleotide polymorphisms; OR, odds ratio; CI, confidence interval.

Subsequently, we conducted MR analyses for each exposure (i.e., gut microbiota) and outcome (i.e., appendicitis), investigating potential causal relationships through five distinct methodologies, namely Inverse Variance Weighted (IVW), MR Egger, weighted median, simple mode, and weighted mode. Employing the IVW method, we identified 11 gut bacteria taxa with potential causal associations with appendicitis. Odds ratios (ORs) were employed to denote the relationship between increased gut bacteria abundance and the risk of appendicitis. The IVW analysis unveiled the following associations: ①At the class level, an increment in Deltaproteobacteria abundance (OR 0.87; 95% CI 0.77-0.98; *p*=0.028) exhibited a negative correlation with appendicitis risk. ②At the family level, augmented levels of Christensenellaceae (OR 0.88; 95% CI 0.79-0.98; *p*=0.016) and Desulfovibrionaceae (OR 0.86; 95% CI 0.75-0.98; *p*=0.026) were negatively associated with appendicitis risk, whereas Family XIII (OR 1.15; 95% CI 1.00-1.32; *p*=0.043) displayed a positive correlation with appendicitis risk. ③At the genus level, increased abundances of Eubacterium ruminantium group (OR 0.92; 95% CI 0.86-0.98; *p*=0.014), Lachnospiraceae NK4A136 group (OR 0.86; 95% CI 0.78-0.95; *p*=0.003), and Methanobrevibacter (OR 0.89; 95% CI 0.80-0.98; *p*=0.018) were protective against appendicitis. Conversely, elevated levels of Howardella (OR 1.13; 95% CI 1.05-1.22; *p*=0.001) and Veillonella (OR 1.12; 95% CI 1.00-1.26; *p*=0.045) posed a risk for appendicitis. ④At the order level, an increase in the abundance of Desulfovibrionales (OR 0.86; 95% CI 0.76-0.98; p=0.027) was negatively correlated with appendicitis. ⑤At the phylum level, an elevation in Euryarchaeota abundance (OR 0.91; 95% CI 0.86-0.97; *p*=0.004) was negatively associated with appendicitis (see **Figure 2**, **Table 1**).

**Table 1:** MR estimates for the association between gut microbiota and appendicitis (*p*<1×10^-5^).

Level	Microbiota	SNPs	Methods	Beta	OR (95% CI)	*p* value
**Class**	Deltaproteobacteria	14	MR Egger	-0.12	0.89(0.62,1.26)	0.511
			Weighted median	-0.08	0.92(0.79,1.08)	0.319
			Inverse variance weighted	-0.14	0.87(0.77,0.98)	0.028
			Simple mode	-0.02	0.98(0.74,1.30)	0.891
			Weighted mode	-0.04	0.96(0.73,1.25)	0.759
**Family**	Christensenellaceae	12	MR Egger	-0.12	0.88(0.72,1.08)	0.253
			Weighted median	-0.11	0.89(0.76,1.04)	0.143
			Inverse variance weighted	-0.13	0.88(0.79,0.98)	0.016
			Simple mode	-0.15	0.86(0.70,1.06)	0.189
			Weighted mode	-0.11	0.90(0.76,1.06)	0.224
**Family**	Desulfovibrionaceae	12	MR Egger	-0.09	0.91(0.63,1.31)	0.829
			Weighted median	-0.08	0.92(0.78,1.09)	0.052
			Inverse variance weighted	-0.16	0.86(0.75,0.98)	0.013
			Simple mode	-0.02	0.98(0.73,1.31)	0.188
			Weighted mode	-0.06	0.94(0.73,1.20)	0.712
**Family**	Family XIII	12	MR Egger	0.20	1.23(0.66,2.27)	0.532
			Weighted median	0.18	1.20(0.99,1.45)	0.057
			Inverse variance weighted	0.14	1.15(1.00,1.32)	0.043
			Simple mode	0.27	1.31(0.92,1.87)	0.169
			Weighted mode	0.26	1.29(0.92,1.82)	0.171
**Genus**	Eubacteriumruminantiumgroup	19	MR Egger	0.07	1.07(0.86,1.33)	0.567
			Weighted median	-0.04	0.96(0.87,1.06)	0.395
			Inverse variance weighted	-0.08	0.92(0.86,0.98)	0.014
			Simple mode	-0.01	0.99(0.84,1.18)	0.937
			Weighted mode	-0.01	0.99(0.85,1.16)	0.896
**Genus**	Howardella	10	MR Egger	0.34	1.40(1.06,1.85)	0.045
			Weighted median	0.13	1.14(1.04,1.25)	0.007
			Inverse variance weighted	0.13	1.13(1.05,1.22)	0.001
			Simple mode	0.06	1.06(0.89,1.25)	0.529
			Weighted mode	0.14	1.15(0.98,1.36)	0.127
**Genus**	Lachnospiraceae NK4A136 group	16	MR Egger	-0.17	0.84(0.69,1.03)	0.115
			Weighted median	-0.12	0.88(0.77,1.01)	0.076
			Inverse variance weighted	-0.15	0.86(0.78,0.95)	0.003
			Simple mode	-0.01	0.99(0.77,1.26)	0.907
			Weighted mode	-0.14	0.87(0.72,1.04)	0.143
**Genus**	Methanobrevibacter	8	MR Egger	-0.26	0.77(0.51,1.16)	0.256
			Weighted median	-0.11	0.89(0.80,1.00)	0.047
			Inverse variance weighted	-0.12	0.89(0.80,0.98)	0.018
			Simple mode	-0.12	0.89(0.72,1.09)	0.287
			Weighted mode	-0.13	0.88(0.72,1.06)	0.215
**Genus**	Veillonella	9	MR Egger	0.55	1.73(0.58,5.14)	0.358
			Weighted median	0.09	1.09(0.94,1.27)	0.268
			Inverse variance weighted	0.12	1.12(1.00,1.26)	0.045
			Simple mode	0.02	1.02(0.80,1.31)	0.853
			Weighted mode	0.03	1.03(0.81,1.30)	0.823
**Order**	Desulfovibrionales	13	MR Egger	-0.11	0.89(0.63,1.27)	0.535
			Weighted median	-0.08	0.93(0.79,1.09)	0.348
			Inverse variance weighted	-0.15	0.86(0.76,0.98)	0.027
			Simple mode	-0.01	0.99(0.75,1.30)	0.920
			Weighted mode	-0.04	0.96(0.74,1.24)	0.761
**Phylum**	Euryarchaeota	13	MR Egger	-0.06	0.95(0.72,1.24)	0.695
			Weighted median	-0.10	0.91(0.83,0.99)	0.037
			Inverse variance weighted	-0.09	0.91(0.86,0.97)	0.004
			Simple mode	-0.12	0.89(0.77,1.03)	0.142
			Weighted mode	-0.09	0.92(0.79,1.06)	0.276

The outcomes of additional analytical methods are presented in **Table 1**, and the scatter plot illustrates potential causal relationships between the gut microbiota and appendicitis. Differently colored lines signify various MR methodologies, including Inverse Variance Weighted (IVW), weighted median, MR-Egger, weighted mode, and simple mode, with each method estimating the causal effects of the gut microbiota on appendicitis (see **Supplementary Figure 2**). The slope value, equivalent to the b value calculated by the five methods, signifies the causal effect of the gut microbiota on appendicitis. A larger absolute slope value denotes a more substantial causal effect. A positive slope indicates exposure as a risk factor, while a negative slope conveys the opposite.

Through our MR analysis, we have successfully identified a total of 11 potential causal relationships between the gut microbiota and appendicitis. To ensure the reliability of our results, a comprehensive set of sensitivity analyses was meticulously carried out to evaluate the potential impact of heterogeneity and pleiotropy among the instrumental variables (IVs) on our findings.

To investigate potential heterogeneity, Cochran’s Q tests were conducted, with all resulting p-values exceeding 0.05. This signifies that no significant heterogeneity was detected among the selected instrumental variables (IVs). Furthermore, horizontal pleiotropy was rigorously assessed through both the MR-Egger intercept and MR-PRESSO global test, both of which returned p-values greater than 0.05, indicating the absence of significant horizontal pleiotropy (see **Table 2**).

**Table 2:** Evaluation of heterogeneity and directional pleiotropy using different methods.

Level	Microbiota	Heterogeneity	Horizontal pleiotropy	
		Cochran’s Q *p*	MR-Egger intercept *p*	MR-PRESSO global test *p*
**Class**	Deltaproteobacteria	0.21	0.16	0.25
**Family**	Christensenellaceae	0.54	0.45	0.60
**Family**	Desulfovibrionaceae	0.16	0.12	0.20
**Family**	Family XIII	0.38	0.30	0.40
**Genus**	Eubacteriumruminantiumgroup	0.62	0.69	0.64
**Genus**	Howardella	0.26	0.37	0.27
**Genus**	Lachnospiraceae NK4A136 group	0.64	0.57	0.69
**Genus**	Methanobrevibacter	0.17	0.14	0.20
**Genus**	Veillonella	0.70	0.67	0.72
**Order**	Desulfovibrionales	0.19	0.14	0.23
**Phylum**	Euryarchaeota	0.60	0.52	0.63

To further bolster the robustness of our results, we conducted additional analyses. Forest plots and leave-one-out analyses were performed, and they collectively demonstrated that no single SNP exerted a strong influence on our MR analysis, further affirming the resilience of our findings (refer to **Supplementary Figure 3**).

## 4 Discussion

In our Mendelian randomization (MR) investigation, we systematically assessed the potential causal association between the gut microbiota and the risk of appendicitis, employing summary statistics derived from established genome-wide association studies (GWAS) on both gut microbiota composition and appendicitis. Our rigorous analysis pinpointed 11 specific bacterial taxa that exhibit a causal link with appendicitis.

Our findings reveal that an augmentation in the abundance of Deltaproteobacteria, Christensenellaceae, Desulfovibrionaceae, Eubacterium ruminantium group, Lachnospiraceae NK4A136 group, Methanobrevibacter, Desulfovibrionales, and Euryarchaeota exerts a protective effect against appendicitis. Conversely, an increase in the abundance of Family XIII, Howardella, and Veillonella is associated with an elevated risk of developing appendicitis.

Our study underscores the notion that alterations in both the diversity and abundance of the gut microbiota may serve as one of the contributing factors to the onset of appendicitis.

Proteobacteria are present within the human gastrointestinal tract, and their prevalence has been observed to elevate notably in cases of severe acute malnutrition among children. Additionally, prior research has documented an escalation in Proteobacteria levels in instances of complicated appendicitis (The et al., 2019). Oh, S.J. et al. additionally proposed that, in comparison to individuals without appendicitis, there is an augmented relative abundance of Alphaproteobacteria and Epsilonproteobacteria, both of which fall under the phylum Proteobacteria, in cases of acute appendicitis. Furthermore, their findings suggest that Campylobacter jejuni could potentially serve as a significant etiological factor in the development of acute appendicitis (Oh et al., 2020). This underscores a significant correlation between specific bacteria within the Proteobacteria phylum and appendicitis, which aligns with our study’s findings. However, in contrast to our investigation, they did not establish a potential causal relationship between Alphaproteobacteria, Epsilonproteobacteria, Campylobacter jejuni, and appendicitis. These results suggest that the heightened abundance of Alphaproteobacteria, Epsilonproteobacteria, and Campylobacter jejuni may arise as a consequence rather than a causative factor in appendicitis. Conversely, the increased prevalence of Deltaproteobacteria, Desulfovibrionales, and Desulfovibrionaceae may serve as preventive factors against the development of appendicitis.

Previous studies have suggested a decrease in the abundance and diversity of Firmicutes in patients with appendicitis (Peeters et al., 2019), a pattern that aligns with our own research findings. However, it remains unresolved whether the reduction in Firmicutes abundance precedes the development of appendicitis or if appendicitis itself triggers a decline in Firmicutes abundance. Our Mendelian randomization (MR) analysis revealed a noteworthy inverse correlation between the heightened abundance of Christensenellaceae, Eubacterium ruminantium group, and Lachnospiraceae NK4A136 group, all constituents of the Clostridia class within the Firmicutes phylum, and the incidence of appendicitis. Particularly, the *p*-value associated with Lachnospiraceae NK4A136 group was less than 0.01, signifying a significant relationship between the decline in Lachnospiraceae NK4A136 group abundance and the onset of appendicitis. These outcomes strongly imply that the decrease in the abundance of these bacterial taxa may serve as a potential causative factor rather than a consequence of appendicitis. Consequently, augmenting the abundance of these bacteria holds promise as a preventive measure against appendicitis.

To date, there has been a dearth of evidence associating Euryarchaeota with appendicitis, with the exception of the detection of methanogens in periappendiceal abscesses as documented in the case report by K Djemai et al (Djemai et al., 2021). Methanogens, which fall under the Euryarchaeota phylum, encompass organisms such as Methanobrevibacter. This observation implies a plausible association between Euryarchaeota, particularly Methanobrevibacter, and appendicitis. Our study further extends these findings by demonstrating that an increase in the abundance of Euryarchaeota and Methanobrevibacter is linked to a reduced risk of appendicitis, indicating that a decline in the prevalence of these bacteria may indeed pose a risk factor for the development of appendicitis. Moreover, the *p*-value associated with Euryarchaeota was 0.004, significantly lower than 0.01, underscoring a strong and close causal relationship between Euryarchaeota and appendicitis.

Within our investigation, we also unearthed a positive correlation between Family XIII, classified under Bacteroidia in the Bacteroidetes phylum, and Veillonella, a member of Negativicutes within the Firmicutes phylum, in relation to appendicitis. Remarkably, as of now, no clinical studies have documented alterations in the abundance or diversity of these bacteria among appendicitis patients. Our study tentatively posits that an elevation in the prevalence of these bacteria might contribute to the development of appendicitis. Based on the outcomes of our research, there emerges a prospect of preventing and managing appendicitis through the manipulation of gut microbiota abundance and diversity. However, it is imperative to underscore that further extensive research is warranted in this regard.

This study represents the inaugural endeavor in employing Mendelian randomization (MR) analysis to probe the causal influence of gut microbiota on appendicitis. In contrast to conventional observational studies, which are susceptible to confounding factors and reverse causality, our investigation furnishes results of heightened reliability. The identification of bacteria with established causal links to appendicitis offers novel and invaluable strategies for the prevention and treatment of appendicitis mediated by the gut microbiota.

Furthermore, the gut microbiota-associated single nucleotide polymorphisms (SNPs) employed in this study stem from the most extensive genome-wide association study (GWAS) meta-analysis conducted to date, affirming the robustness of the instrumental variables (IVs) incorporated into our research. The substantial sample size, coupled with the application of diverse sensitivity analyses, ensures the resilience and validity of our study findings.

Nonetheless, it is imperative to acknowledge that, while our study identifies a causal relationship between the gut microbiota and appendicitis, the potential influence of appendicitis on the composition and diversity of the gut microbiota cannot be entirely discounted. Further research is necessitated to delve into the intricate interplay between these factors.

Furthermore, it is noteworthy that the gut microbiota GWAS data utilized in this study predominantly originate from individuals of European ancestry, with a limited representation of non-European ancestry data. Simultaneously, the appendicitis GWAS data exclusively consist of individuals of European ancestry. This demographic skew could introduce bias into our study and restrict the applicability of our findings to other populations.

While our investigation uncovers a plausible causal connection between the gut microbiota and appendicitis, it should be noted that direct mechanistic research is lacking to substantiate our study’s outcomes. Consequently, there exists a pressing need to embark on research elucidating the mechanistic impact of the gut microbiota on appendicitis, drawing from the findings concerning the 11 specific gut bacteria identified in this study. This endeavor holds promise for a more comprehensive understanding of the etiology of appendicitis and the development of innovative preventive and therapeutic strategies.

## 5 Conclusion

This pioneering study, employing Mendelian randomization (MR) analysis, furnishes genetic evidence substantiating the causal influence of gut microbiota on appendicitis. The discerned gut microbiota, whether beneficial or deleterious in the context of appendicitis, could potentially present novel and invaluable avenues for the prevention and treatment of appendicitis through interventions targeting the gut microbiota.

## Data availability statement

The datasets presented in this study can be found in online repositories. The names of the repository/repositories and accession number(s) can be found in the article/**Supplementary Material**.

## Author contributions

ZW: Data curation, Writing – original draft, Writing – review & editing. LB: Writing – review & editing. LW: Writing – review & editing. QZ: Writing – review & editing. QF: Writing – review & editing. JZ: Writing – review & editing. ZL: Writing – review & editing. YW: Writing – original draft, Writing – review & editing.
