# Supplementary material for: Causal effects of gut microbiota on appendicitis: a two-sample Mendelian randomization study
Source: Front Cell Infect Microbiol. 2023 Dec 15;13:1320992. doi: 10.3389/fcimb.2023.1320992 (PMC10757326; doi:10.3389/fcimb.2023.1320992)
Supplement: Supplementary file 1 [file DataSheet_1.pdf]

Supplementary Material

1 Supplementary Figures

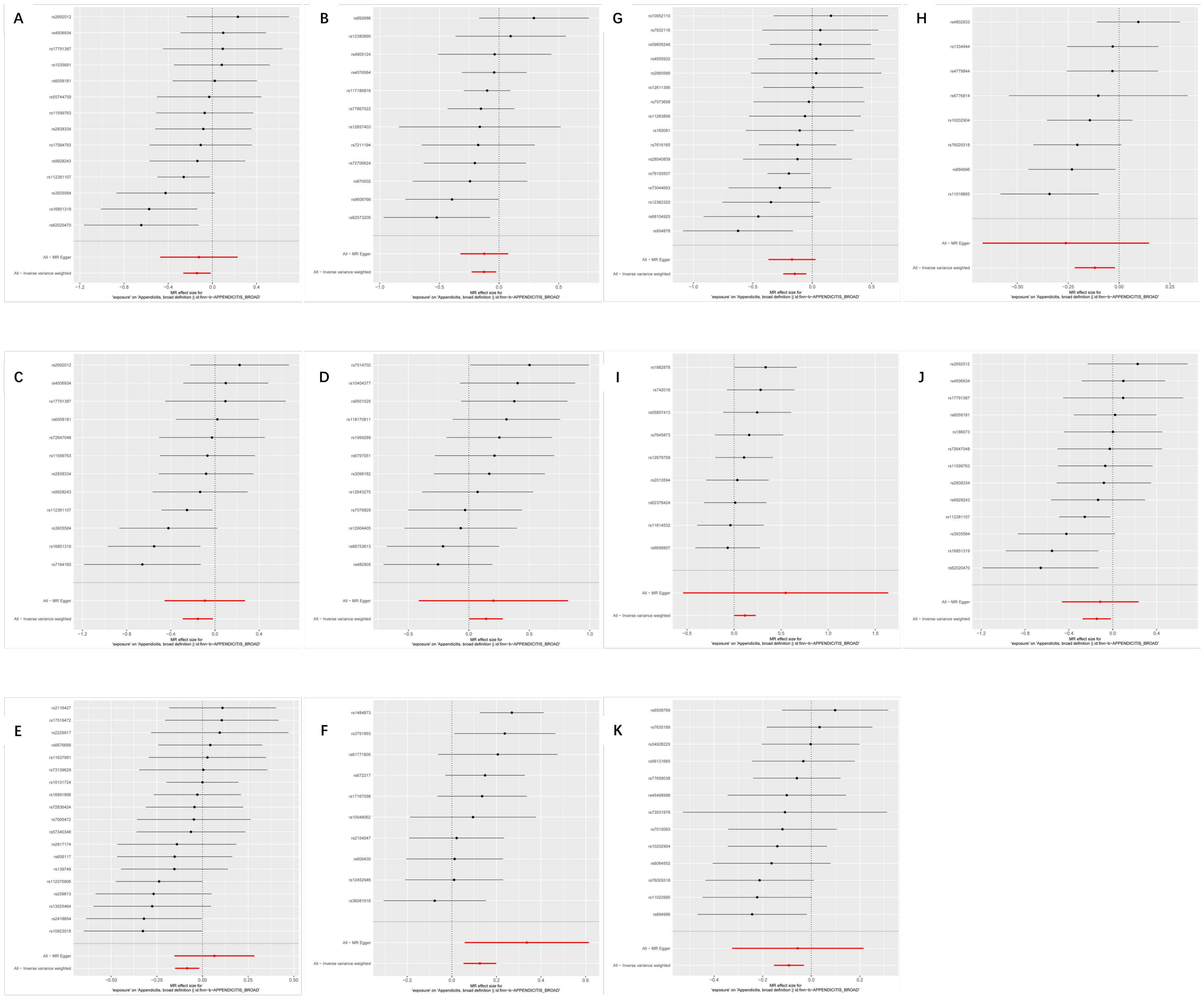

Supplementary Figure 1. Forest plots of the causal effects of gut microbiota on the risk of appendicitis. **(A)** Deltaproteobacteria; **(B)** Christensenellaceae; **(C)** Desulfovibrionaceae; **(D)** Family XIII; **(E)** Eubacteriumruminantiumgroup; **(F)** Howardella; **(G)** LachnospiraceaeNK4A136group; **(H)** Methanobrevibacter;**(I)** Veillonella;**(J)** Desulfovibrionales;**(K)** Euryarchaeota.

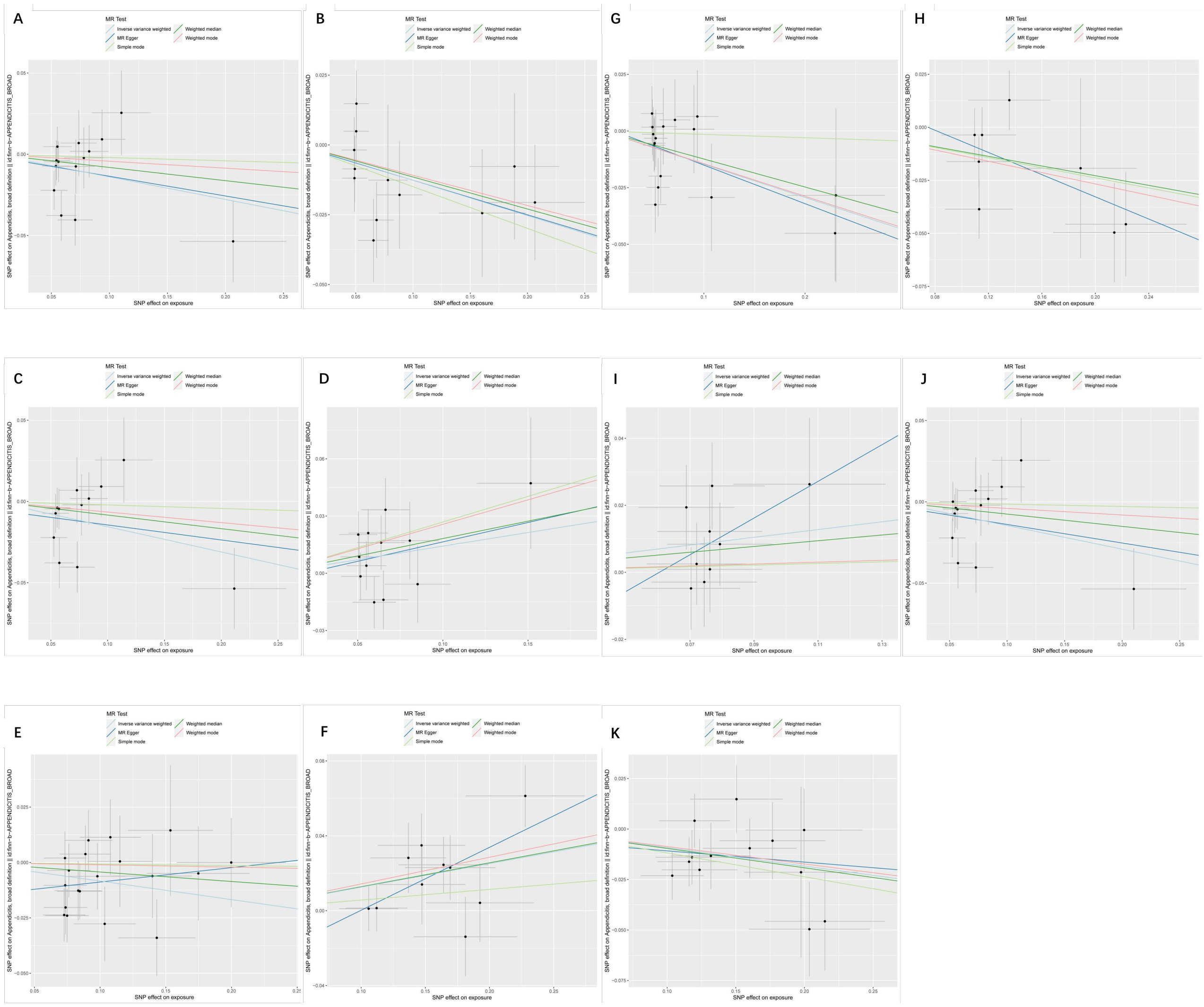

Supplementary Figure 2. Scatter plots of the causal effect of gut microbiota on the risk of appendicitis. **(A)** Deltaproteobacteria; **(B)** Christensenellaceae; **(C)** Desulfovibrionaceae; **(D)** Family XIII; **(E)** Eubacteriumruminantiumgroup; **(F)** Howardella; **(G)** LachnospiraceaeNK4A136group; **(H)** Methanobrevibacter;**(I)** Veillonella;**(J)** Desulfovibrionales;**(K)** Euryarchaeota.

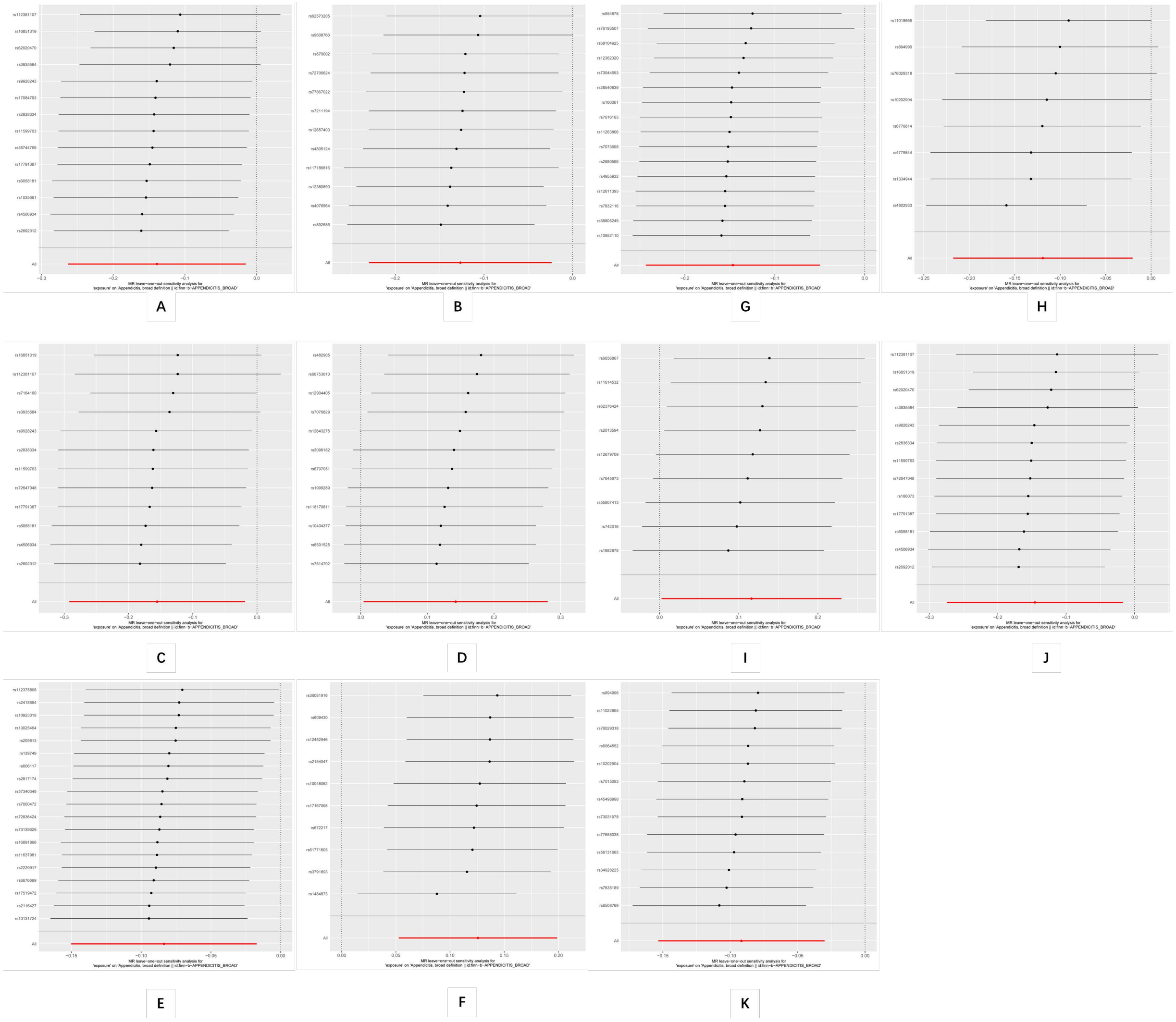

Supplementary Figure 3. Leave-one-out sensitivity analyses of the causal effects of gut microbiota on the risk of appendicitis. **(A)** Deltaproteobacteria; **(B)** Christensenellaceae; **(C)** Desulfovibrionaceae; **(D)** Family XIII; **(E)** Eubacteriumruminantiumgroup; **(F)** Howardella; **(G)** LachnospiraceaeNK4A136group; **(H)** Methanobrevibacter;**(I)** Veillonella;**(J)** Desulfovibrionales;**(K)** Euryarchaeota.

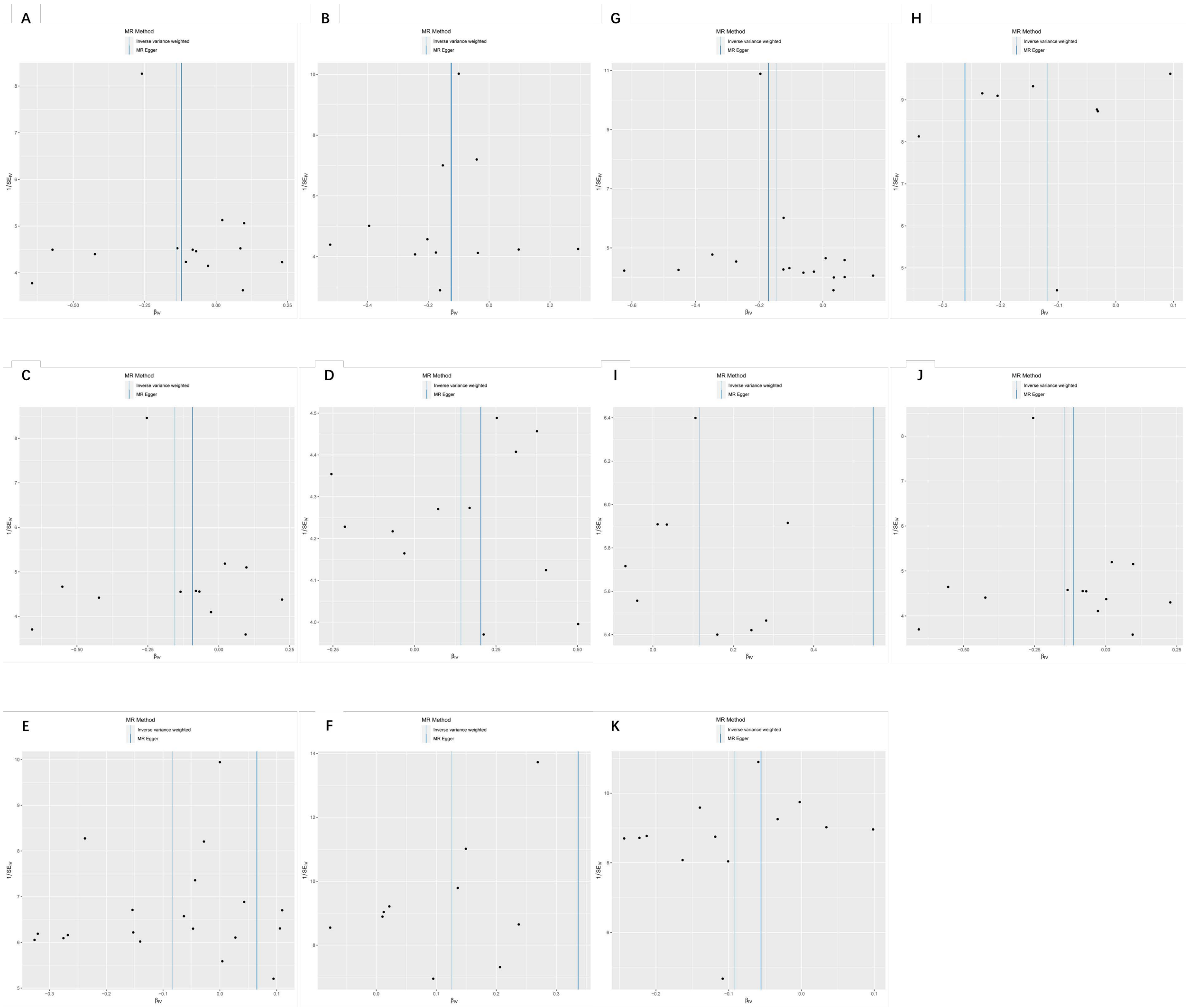

Supplementary Figure 4. Funnel-plot of the causal effects of gut microbiota on the risk of appendicitis. **(A)** Deltaproteobacteria; **(B)** Christensenellaceae; **(C)** Desulfovibrionaceae; **(D)** Family XIII; **(E)** Eubacteriumruminantiumgroup; **(F)** Howardella; **(G)** LachnospiraceaeNK4A136group; **(H)** Methanobrevibacter;**(I)** Veillonella;**(J)** Desulfovibrionales;**(K)** Euryarchaeota.

2 Supplementary Tables

Supplementary Table 1.

SNP	other_allele.exposure	effect_allele exposure	beta.exposure	se.exposure	pval.exposure	samplesize.exposure	R2	F
**rs10048062**	T	C	-0.14735	0.033653	8.59E-06	3800	0.005023	19.1617
**rs10131724**	C	A	-0.19983	0.041458	2.39E-06	5071	0.002993	23.22781
**rs10202904**	G	T	-0.11281	0.023911	3.09E-06	3583	0.006529	22.24668
**rs1035691**	G	A	-0.05518	0.01216	9.65E-06	15168	0.001362	20.58557
**rs10404377**	A	C	-0.05032	0.011165	6.99E-06	16269	0.001247	20.30863
**rs10923018**	A	G	0.072644	0.016092	6.80E-06	7739	0.002626	20.37243
**rs10952110**	T	G	0.048771	0.010961	9.08E-06	16764	0.001179	19.79403
**rs11018665**	T	A	0.113013	0.025434	7.03E-06	3586	0.005795	19.73161
**rs11022995**	A	G	-0.10373	0.022889	7.73E-06	3864	0.005291	20.52733
**rs112375806**	A	T	0.143141	0.029376	5.82E-06	7123	0.003059	23.73769
**rs112381107**	T	C	0.206594	0.045691	4.63E-06	4457	0.001352	20.44188
**rs11263806**	G	A	-0.05246	0.011676	5.07E-06	17236	0.001203	20.1863
**rs11599763**	C	T	-0.05442	0.01174	3.94E-06	15168	0.001421	21.48714
**rs11614532**	C	G	0.074454	0.016521	7.13E-06	8411	0.003271	20.30198
**rs11637981**	T	G	-0.07326	0.016089	5.44E-06	7718	0.002672	20.72768
**rs117186816**	A	G	-0.20648	0.043636	6.52E-06	4310	0.001328	22.38677
**rs118170811**	G	A	0.151619	0.031711	1.80E-06	9645	0.001403	22.8575
**rs12362320**	C	G	0.057317	0.011547	8.04E-07	16764	0.001468	24.63778
**rs12380890**	G	A	-0.0504	0.011105	5.78E-06	16846	0.001222	20.5942
**rs12452946**	G	A	-0.10583	0.022894	3.80E-06	3800	0.005595	21.35835
**rs12611395**	G	A	-0.09025	0.019965	5.83E-06	16486	0.001218	20.43264
**rs12643275**	T	A	-0.05509	0.011647	2.42E-06	16269	0.001373	22.36895
**rs12657403**	G	A	0.078108	0.017189	5.59E-06	16245	0.001225	20.64689
**rs12679709**	G	C	-0.07935	0.016482	1.78E-06	9290	0.003731	23.17095
**rs12904405**	G	A	-0.08519	0.019269	7.67E-06	16643	0.0012	19.54379
**rs13025464**	C	T	-0.07371	0.016379	6.97E-06	7738	0.00261	20.24703
**rs1334944**	C	T	0.115197	0.025549	7.61E-06	3586	0.005966	20.31731
**rs139749**	T	C	-0.08454	0.017179	8.59E-07	7314	0.00312	24.21324
**rs1484873**	G	A	-0.22783	0.046335	2.56E-06	3608	0.006326	24.16455
**rs160061**	G	A	0.051383	0.010809	2.12E-06	17234	0.001346	22.59357
**rs16851319**	C	G	-0.07055	0.015085	5.68E-06	15154	0.001447	21.87099
**rs16891896**	A	G	-0.17479	0.039057	2.38E-06	4753	0.002581	20.02176
**rs17084793**	A	G	-0.07108	0.015953	5.69E-06	15164	0.001313	19.84747
**rs17167098**	A	G	-0.16937	0.035207	1.12E-06	3586	0.006056	23.12966
**rs17519472**	T	C	0.107804	0.023398	4.70E-06	7710	0.002735	21.22204
**rs17791387**	G	A	-0.07359	0.015425	1.60E-06	15071	0.001505	22.75772
**rs186073**	C	T	0.052922	0.011854	8.74E-06	14841	0.001314	19.92833
**rs1882878**	G	A	-0.0769	0.016391	2.98E-06	9282	0.003544	22.00249
**rs1999289**	T	A	-0.06374	0.013927	2.54E-06	16269	0.001286	20.94166
**rs2013594**	C	T	-0.07207	0.015515	3.42E-06	9283	0.003474	21.56974
**rs209813**	A	G	-0.10349	0.023639	9.23E-06	7264	0.00247	19.16057
**rs2116427**	G	A	0.091146	0.018235	4.67E-07	7739	0.003218	24.97683
**rs2154047**	A	C	-0.19256	0.042009	9.97E-06	3623	0.005501	20.99899

**rs2229917**	G	A	0.153538	0.032392	2.16E-06	7050	0.002895	22.46145
**rs2418654**	T	C	-0.07489	0.016585	6.17E-06	7314	0.002628	20.38316
**rs2692012**	G	A	0.110335	0.025334	3.14E-06	13104	0.001255	18.96598
**rs2817174**	T	C	-0.07343	0.016369	7.87E-06	7710	0.002594	20.11935
**rs2838334**	A	G	0.056172	0.012412	5.45E-06	15168	0.001354	20.47735
**rs28540839**	C	A	0.050828	0.011059	9.34E-06	16764	0.001258	21.1214
**rs2880566**	C	T	0.059958	0.013469	5.61E-06	17232	0.001181	19.81309
**rs3098182**	T	G	0.050851	0.011014	4.04E-06	16731	0.001309	21.31348
**rs34928225**	C	T	0.19977	0.042542	4.33E-06	3483	0.005679	22.03921
**rs36081916**	C	T	-0.18123	0.040299	4.70E-06	3216	0.005297	20.21378
**rs3791893**	G	A	0.147035	0.034023	9.50E-06	3795	0.004893	18.66677
**rs3935584**	T	C	-0.05235	0.011566	7.50E-06	15166	0.001355	20.48239
**rs4076564**	A	G	-0.18862	0.039209	8.06E-06	3730	0.001373	23.14014
**rs4506934**	T	C	-0.09365	0.02012	3.59E-06	15061	0.001433	21.66273
**rs45498998**	A	G	-0.13188	0.029229	5.32E-06	3850	0.005245	20.34796
**rs4779844**	C	G	0.109639	0.024798	9.28E-06	3585	0.005738	19.53629
**rs4802933**	G	A	-0.13563	0.030814	9.74E-06	3586	0.005687	19.3621
**rs4805124**	G	C	0.048651	0.010907	8.36E-06	16847	0.00118	19.8949
**rs482905**	T	G	0.059653	0.012717	3.72E-06	16737	0.001351	22.00075
**rs4955932**	C	T	-0.04923	0.010939	7.05E-06	17236	0.001207	20.2507
**rs55744759**	G	A	-0.07797	0.017074	7.31E-06	15168	0.001379	20.85103
**rs55807413**	G	A	0.107329	0.023763	5.51E-06	8610	0.003285	20.39302
**rs56131665**	A	G	0.176759	0.038579	5.30E-06	3736	0.005408	20.98184
**rs57340348**	C	T	-0.09794	0.021217	4.93E-06	7314	0.002746	21.30505
**rs59805249**	C	T	0.093616	0.020798	9.45E-06	15195	0.001207	20.25804
**rs6058181**	T	C	0.082572	0.016596	3.40E-07	15100	0.001637	24.75176
**rs606117**	G	A	0.083324	0.018056	4.82E-06	7263	0.002744	21.29062
**rs6064552**	C	T	-0.12361	0.027575	9.34E-06	3775	0.005178	20.08475
**rs609430**	G	T	-0.11205	0.023933	3.34E-06	3798	0.005738	21.90648
**rs61771805**	T	A	-0.13678	0.029681	4.03E-06	3612	0.00556	21.225
**rs62020470**	G	A	-0.05854	0.012936	4.85E-06	15168	0.001354	20.4774
**rs62376424**	T	C	-0.07622	0.01635	3.65E-06	8999	0.003499	21.72625
**rs62573205**	A	G	-0.06548	0.01335	1.49E-06	16838	0.001427	24.05411
**rs6501525**	G	A	0.056156	0.011559	1.24E-06	16269	0.001449	23.60031
**rs6508769**	C	T	0.150529	0.033709	8.12E-06	3483	0.005138	19.93055
**rs6656807**	G	A	0.070306	0.015395	5.50E-06	8999	0.003358	20.84806
**rs66753613**	A	G	0.065114	0.014425	8.08E-06	16269	0.001251	20.37278
**rs6676699**	T	G	-0.08881	0.019645	6.38E-06	7708	0.002634	20.43351
**rs672217**	A	G	0.164146	0.034999	3.52E-06	3611	0.005758	21.98428
**rs6776814**	C	T	-0.18896	0.041991	8.05E-06	3283	0.005943	20.2378
**rs6797051**	T	C	-0.0806	0.017136	4.89E-06	16737	0.001358	22.11873
**rs68104925**	C	T	-0.05491	0.011538	2.37E-06	17227	0.001349	22.64426
**rs7000472**	G	A	-0.07623	0.016523	4.07E-06	7739	0.002743	21.27846
**rs7015093**	A	G	-0.11811	0.026439	7.20E-06	3855	0.005142	19.94657
**rs7073658**	G	T	-0.04996	0.010969	5.27E-06	17236	0.001236	20.7431
**rs7076829**	A	T	-0.05164	0.011231	4.76E-06	16269	0.001298	21.13732
**rs7164160**	T	A	-0.05745	0.012947	7.34E-06	15142	0.001304	19.68459

**rs7211194**	T	C	0.049209	0.011092	9.94E-06	16436	0.001168	19.68126
**rs72647048**	C	T	-0.07701	0.017089	9.61E-06	15142	0.001345	20.30433
**rs72706624**	T	G	0.088263	0.019757	6.38E-06	14869	0.001184	19.95439
**rs72836424**	T	C	-0.13982	0.030069	2.62E-06	7314	0.002786	21.61812
**rs73031978**	G	C	-0.19752	0.04145	2.74E-06	3501	0.005847	22.69687
**rs73044693**	G	A	-0.10758	0.022988	3.57E-06	12773	0.001305	21.89734
**rs73139629**	C	A	-0.1151	0.024791	5.36E-06	7314	0.002778	21.54945
**rs742016**	G	A	-0.06886	0.014977	4.66E-06	9279	0.003404	21.13063
**rs7514702**	C	T	-0.06632	0.014163	3.92E-06	16737	0.001346	21.92803
**rs76029318**	C	T	0.222849	0.045432	1.08E-06	3387	0.007054	24.04594
**rs7616165**	T	G	-0.23054	0.048346	2.77E-06	4094	0.001355	22.73626
**rs76193507**	G	A	-0.22973	0.049978	2.93E-06	3578	0.001259	21.12682
**rs7635189**	A	G	0.119983	0.025936	4.64E-06	3483	0.005513	21.39066
**rs7645873**	T	A	0.076139	0.016423	3.12E-06	9286	0.003461	21.48704
**rs77658038**	C	A	-0.16016	0.034086	4.75E-06	3810	0.005686	22.06596
**rs77867022**	T	G	-0.16044	0.037959	8.82E-06	4033	0.00106	17.86346
**rs7832116**	G	A	-0.07148	0.01517	3.57E-06	17219	0.001322	22.19601
**rs870002**	T	C	0.048895	0.010982	6.54E-06	16847	0.001176	19.82171
**rs892686**	G	A	0.050611	0.010965	4.12E-06	16847	0.001264	21.30153
**rs894996**	A	C	0.214213	0.045604	3.82E-06	3479	0.006472	22.05051
**rs954878**	G	A	-0.05207	0.010908	1.78E-06	17227	0.001357	22.77941
**rs9608766**	C	T	-0.06821	0.01496	3.33E-06	16185	0.001233	20.78868
**rs9928243**	A	C	-0.05385	0.011772	5.02E-06	15167	0.001384	20.9199
